# The Yin and Yang of GABAergic and Glutamatergic Synaptic Plasticity: Opposites in Balance by Crosstalking Mechanisms

**DOI:** 10.3389/fnsyn.2022.911020

**Published:** 2022-05-19

**Authors:** Caitlyn A. Chapman, Jessica L. Nuwer, Tija C. Jacob

**Affiliations:** Department of Pharmacology and Chemical Biology, University of Pittsburgh School of Medicine, Pittsburgh, PA, United States

**Keywords:** synaptic plasticity, GABA receptor (GABA-R), glutamate, NMDA receptor, LTP (long term potentiation), LTD (long term depression), calcium, activity

## Abstract

Synaptic plasticity is a critical process that regulates neuronal activity by allowing neurons to adjust their synaptic strength in response to changes in activity. Despite the high proximity of excitatory glutamatergic and inhibitory GABAergic postsynaptic zones and their functional integration within dendritic regions, concurrent plasticity has historically been underassessed. Growing evidence for pathological disruptions in the excitation and inhibition (E/I) balance in neurological and neurodevelopmental disorders indicates the need for an improved, more “holistic” understanding of synaptic interplay. There continues to be a long-standing focus on the persistent strengthening of excitation (excitatory long-term potentiation; eLTP) and its role in learning and memory, although the importance of inhibitory long-term potentiation (iLTP) and depression (iLTD) has become increasingly apparent. Emerging evidence further points to a dynamic dialogue between excitatory and inhibitory synapses, but much remains to be understood regarding the mechanisms and extent of this exchange. In this mini-review, we explore the role calcium signaling and synaptic crosstalk play in regulating postsynaptic plasticity and neuronal excitability. We examine current knowledge on GABAergic and glutamatergic synapse responses to perturbances in activity, with a focus on postsynaptic plasticity induced by short-term pharmacological treatments which act to either enhance or reduce neuronal excitability via ionotropic receptor regulation in neuronal culture. To delve deeper into potential mechanisms of synaptic crosstalk, we discuss the influence of synaptic activity on key regulatory proteins, including kinases, phosphatases, and synaptic structural/scaffolding proteins. Finally, we briefly suggest avenues for future research to better understand the crosstalk between glutamatergic and GABAergic synapses.

## Introduction

Ligand-gated ion channel GABA type A receptors (GABA_A_Rs) mediate the majority of fast inhibition in the central nervous system, while glutamatergic AMPA receptors (AMPARs) and NMDA receptors (NMDARs) collectively mediate fast excitatory neurotransmission. NMDARs particularly play a unique role in synaptic plasticity due to high calcium permeability and voltage-dependent Mg^2+^ block typically relieved by AMPAR-mediated depolarization. Slow inhibition and excitation are generated by G protein-coupled, GABA type B (GABA_B_Rs) and metabotropic glutamate receptors (mGluRs), respectively. The concerted action of these receptors balances neuronal excitability. A close and coordinated spatial relationship between glutamatergic and GABAergic synapses on dendrites (Megías et al., 2001; Bleckert et al., 2013; Iascone et al., 2020), sometimes as near as on the same spine (Chen et al., 2012), facilitates synaptic input integration, dynamic calcium regulation, synaptic crosstalk, and coregulation.

Synaptic plasticity describes the ability of synapses to adapt their relative strength based on the overall level of activity or specific activity patterns, often by dynamic regulation of receptor-synaptic scaffold interactions or through trafficking. During development, it is heavily involved in dendritic growth, synaptogenesis, and the formation of neural circuits (reviewed in Akgül and McBain, 2016; Ismail et al., 2017; Jenks et al., 2021). In mature neurons, synaptic plasticity is responsible for synapse remodeling during experience. Genetic mutations or pathology leading to altered excitatory or inhibitory neurotransmission or impaired synaptogenesis typically result in deficits in synaptic plasticity, a common feature in neurodevelopmental and neurological disorders (Rudolph and Möhler, 2014; Mele et al., 2019), including autism (Hansel, 2019; Sohal and Rubenstein, 2019), down syndrome (Galdzicki et al., 2001; Schulz et al., 2019), schizophrenia (Lewis and Moghaddam, 2006; Gao and Penzes, 2015), epilepsy (Needs et al., 2019), and neurodegenerative disorders (Smith-Dijak et al., 2019; Bi et al., 2020). Uncovering the mechanisms regulating synaptic plasticity will help illuminate how disruptions in GABAergic and glutamatergic function influence the pathophysiology of these disorders, identify new therapeutic targets, and reveal potential impacts of pharmacologically targeting these receptors.

Homeostatic and Hebbian plasticity constitute two major forms of activity-dependent regulation of synaptic transmission (Kavalali and Monteggia, 2020; reviewed in Galanis and Vlachos, 2020). During Hebbian plasticity, synapses rapidly respond to a stimulus in the same direction as the applied stimulus. These Hebbian mechanisms typically result in a persistent strengthening or weakening of synapses, termed long-term potentiation (LTP) and long-term depression (LTD), respectively. NMDAR-dependent LTP of excitatory synapses in the hippocampus is the most studied experimental model for investigating the synaptic basis of plasticity, learning, and memory in vertebrates (Collingridge et al., 1983; Bliss and Collingridge, 1993; Martin et al., 2000; Neves et al., 2008; Stuchlik, 2014). On the other hand, during homeostatic plasticity, synapses respond in the opposite direction and on a slower timescale from the applied stimulus, compensating for the shift in activity. For example, 24-h treatment with the GABA_A_R competitive antagonist bicuculline overall upregulates inhibition, increasing GABA_A_R surface clustering and reducing miniature excitatory postsynaptic currents (mEPSCs) (Turrigiano et al., 1998; Pribiag et al., 2014). Although the importance of inhibitory synapse plasticity has become more apparent in recent years, research has largely continued to divide focus between excitation or inhibition, studying one or the other in isolation. However, increasing evidence indicates a coordination between GABAergic and glutamatergic synapses to maintain an optimal orchestrated balance of neuronal activity (Higley, 2014; Jedlicka et al., 2018; Chiu et al., 2019). Activity-dependent functioning of proteins in calcium-dependent signaling pathways appears to be the primary mechanism for synaptic crosstalk during plasticity, with important examples including voltage-gated calcium channels; the kinases CaMKII and PKC; the phosphatase calcineurin; and the protease calpain. However, there is a lack of sufficient understanding of these mechanisms. Further studies which simultaneously investigate excitatory and inhibitory synaptic responses under various plasticity-inducing protocols are needed, with a particular focus at the receptor level and mechanisms of crosstalk. Pharmacological manipulation provides valuable insight into the plasticity of synaptic ionotropic receptors under differential states of activity. In this mini-review, we focus primarily on brief to intermediate (30-min–48-h) pharmacologically-induced postsynaptic plasticity of GABAergic and glutamatergic synapses studied using the long-standing model of rodent neuron cultures and discuss potential key proteins involved in mediating synaptic crosstalk.

## GABA Type a Receptor Synaptic Plasticity Induced by Pharmacological Treatments

Receptor trafficking, particularly lateral diffusion between synaptic and extrasynaptic sites, rapidly regulates excitatory or inhibitory synaptic strength (reviewed in Bard and Groc, 2011; Ladépêche et al., 2014; Petrini and Barberis, 2014; Maynard and Triller, 2019). The postsynaptic scaffolds gephyrin and PSD95 help tether ionotropic receptors at inhibitory and excitatory synapses, respectively, while diffusion to extrasynaptic regions facilitates receptor internalization (Blanpied et al., 2002; Rácz et al., 2004; Thomas et al., 2005; Bogdanov et al., 2006). As discussed below, pharmacologically altering neuronal activity influences receptor trafficking and synaptic localization to dynamically modulate synaptic strength.

### Acute Changes in Neuronal Activity

Overall, moderate-to-high increases in neuronal activity on an acute timescale induce iLTD through enhanced GABA_A_R diffusion and reduced postsynaptic clustering. For example, dramatically increasing activity with the potassium channel blocker 4-aminopyridine (4AP, 100 μM) or NMDAR stimulation (NMDA + co-agonist glycine + tetrodotoxin) for <30-min immediately destabilizes inhibitory synapses via reduced synaptic GABA_A_R confinement and decreases the amplitude of miniature inhibitory postsynaptic currents (mIPSCs) (Niwa et al., 2012; Lévi et al., 2015). Pharmacological receptor stabilization by the benzodiazepine (BZD) diazepam (DZP), a GABA_A_R positive allosteric modulator, prevents the 4AP-induced mobility increase (Lévi et al., 2015). Enhanced GABA_A_R diffusion is similarly observed during NMDAR activation by co-application of glutamate and glycine (Muir et al., 2010) or when GABA_A_R activity is reduced by the negative allosteric modulator (NAM), methyl-6,7-dimethoxyl-4-ethyl- β-carboline-3-carboxylate (DMCM) (Lévi et al., 2015). These rapid effects rely on calcineurin activation and phosphorylation of the γ2 subunit present in the majority of synaptic GABA_A_Rs and appear to be independent of receptor internalization; inhibition of endocytosis with dynasore did not impact the 4AP- or glutamate/glycine-induced effects (Bannai et al., 2009; Muir et al., 2010). In contrast, Saliba et al. (2012) observed a CaMKII-dependent increase in surface β3-GABA_A_R with 30-min 4AP (50 μM) along with enhanced tonic current. eLTD/iLTP protocols with brief, moderate NMDA stimulation (2-min NMDA + AMPAR antagonist CNQX) followed by a short recovery period (10–20 min) also enhanced CaMKII-mediated cell-surface insertion of β2/3-GABA_A_R, increased mIPSC amplitude, and stabilized synapse-specific GABA_A_R (Marsden et al., 2007; Petrini et al., 2014). CaMKII-mediated receptor insertion may also represent a potential mechanism for GABA_A_R recovery in response to dramatic increases in activity. GABA_A_R synaptic cluster loss following brief NMDAR activation is recovered within 40-min via either new receptor insertion or reclustering of existing surface receptors (Muir et al., 2010). Thus, it appears opposing effects on GABA_A_R plasticity may reflect how specific experimental conditions produce localized time-dependent calcium concentration dynamics activating either calcineurin at high [Ca^2+^] or CaMKII at moderate-to-low [Ca^2+^].

Based on these observations, acute pharmacological enhancement of inhibition might be anticipated to promote GABA_A_R synapse stabilization. Contrary to this expectation, 30-min treatment with the GABA_A_R agonist muscimol increases GABA_A_R diffusivity and reduces receptor and scaffold clustering (Gouzer et al., 2014; Brady et al., 2018). Intriguingly, there is an overall rearrangement of distinct populations of receptors, whereby γ2-GABA_A_Rs shift to extrasynaptic sites and non-γ2-GABA_A_Rs increase in the synapse (Brady et al., 2018). These iLTD-like responses to a GABA_A_R agonist are reminiscent to those observed with acute enhancement of excitation. While GABA_A_R agonists destabilize synapses, 30-min application of GABA_A_R antagonists such as gabazine or picrotoxin increase GABA_A_R synaptic prevalence in an iLTP-like fashion (Gouzer et al., 2014). In contrast to muscimol-destabilization of inhibitory synapses, muscimol and DZP co-treatment stabilizes GABAergic synapses. Interestingly, DZP stabilizes receptors independent of activity and calcium (Gouzer et al., 2014), with similar reductions in both muscimol and 4AP-mediated GABA_A_R diffusion. GABA_A_R subtypes exhibit differential clustering, synaptic localization, drug binding sites, and intracellular protein interactors, thus potentially invoking different plasticity mechanisms. In support of this, the effects of DZP on receptor dynamics was dependent on the gephyrin binding motif (Gouzer et al., 2014).

### Chronic Changes in Neuronal Activity

Compared to changes observed with acute activity modulation, homeostatic synaptic plasticity in response to 24–48-h pharmacological manipulation functions to re-establish neuronal activity balance. For example, 48-h-4AP-enhanced activity increases synaptic gephyrin and α2-GABA_A_Rs with concurrent reduced α2-GABA_A_R diffusion (Battaglia et al., 2018). Additionally, global depletion of neuronal activity for 24–48-h with tetrodotoxin (TTX) substantially restructures both glutamatergic and GABAergic synapses. This is characterized by reduced surface/total levels and clustering of the major synaptic γ2-GABA_A_Rs (Gouzer et al., 2014), reduced presynaptic GAD65, and diminished GABAergic neurotransmission (Kilman et al., 2002; Swanwick et al., 2006). Meanwhile, TTX leads to increased mEPSCs, indicating upregulated excitatory transmission (O’Brien et al., 1998; Turrigiano et al., 1998; Watt et al., 2000). The effects of TTX on inhibitory synapses were replicated by pharmacological blockade of either AMPARs by DNQX or NMDARs by APV (Swanwick et al., 2006), implicating a role for these receptors in inhibitory plasticity induction. Furthermore, 24–48-h treatment with the GABA_A_R competitive antagonist bicuculline overall upregulates inhibition by increasing GABA_A_R surface clustering and reducing mEPSCs (Turrigiano et al., 1998; Pribiag et al., 2014). As early as 4-h of bicuculline treatment increases the expression of GAD65 and VGAT presynaptically and α1-GABA_A_R postsynaptically, accompanied by a corresponding functional enhancement of inhibition (Peng et al., 2010). Co-application of the AMPAR antagonist NBQX blocks these responses. Bicuculline-induced increases in surface α5-GABA_A_Rs and tonic inhibition after 24–48 h are also blocked by APV or the GluN2A-preferring antagonist NVP (Wu et al., 2021a). Activity-dependent scaling of inhibition was recently demonstrated in detail using super-resolution microscopy. Specifically, 24-h bicuculline increases the number of subsynaptic domains per synapse and increases the compartment volume each of gephyrin, GABA_A_Rs, and the inhibitory postsynaptic density (Crosby et al., 2019). Such homeostatic responses equilibrate E/I balance throughout prolonged modulation of neuronal excitability.

Despite the intrinsic utility of homeostasis, chronic therapeutic targeting of these receptors can prompt homeostatic responses that are detrimental to maintaining the desired response. For example, BZDs are clinically used in treatment of seizures, anxiety, and insomnia, but tolerance and dependence develop with long-term use. Withdrawal symptoms occur upon discontinuation and are characterized by hallmarks of hyperexcitability, including increased risk of seizures, insomnia, and anxiety. Correspondingly, prolonged (12–48-h) BZD treatment downregulates inhibition through multiple interconnected mechanisms. This includes through reduced transcription of α1 subunits (Foitzick et al., 2020); increased degradation of BZD-sensitive α2 and γ2 subunits (Jacob et al., 2012; Lorenz-Guertin et al., 2019); reduced gephyrin clustering via increased activity of the calcium-activated protease calpain (Vlachos et al., 2013; Lorenz-Guertin et al., 2019); and enhanced surface mobility of γ2-GABA_A_R and increased inhibitory synaptic turnover (Lorenz-Guertin et al., 2019). Together these events likely contribute to a functional reduction of DZP potentiation and inhibition overall (Zeng and Tietz, 1999; Jacob et al., 2012; Vlachos et al., 2013). Moving forward, fundamental studies focused on concurrent basal synaptic plasticity and translational efforts centered on how chronic use of therapeutic agents modifies plasticity at excitatory and inhibitory synapses will be invaluable.

## Interplay Between Glutamatergic and Gabaergic Synapses

Research has generally separated its focus between excitation or inhibition, despite the ever-present, dynamic coordination and integration between both synapse types necessary for orchestrating appropriate neuronal activity. This is largely due to the greater experimental load and range of required reagents, time, and expertise this necessitates. Developmental studies have thus far provided important insight into the interplay between glutamatergic and GABAergic synapses (reviewed in Jenks et al., 2021). However, insufficient research has been aimed at understanding crosstalking mechanisms at the receptor level in mature neurons. Studies which previously investigated both synapse types have often evaluated plasticity responses only on a macroscopic level (population field potentials), which lends difficulty in discerning what underlying changes in excitation and/or inhibition contribute to the net result. Accumulating evidence points to complex mechanisms at play involving a convergence of signaling cascades, facilitating crosstalk between synapse types.

### Effect of Glutamate Receptor Signaling on Inhibitory Synaptic Plasticity

Much of what is known about synaptic crosstalk resulting in inhibitory plasticity is based on excitatory protocols activating NMDARs or mGluRs. Exposure to NMDA or aspartate activates NMDARs and subsequently suppresses GABA_A_R current (Stelzer and Shi, 1994; Chisari et al., 2012) in a GABA concentration-dependent manner (Cong et al., 2011), and *vice versa*—NMDAR currents can be suppressed by GABA_A_R pre-activation (Cong et al., 2011). Recent GluN2A vs. GluN2B NMDAR subtype-specific crosstalk effects were identified; 24-h antagonism of GluN2A in cultured neurons at days *in vitro* 14 (DIV14) with NVP-AAM077 decreased surface α5-GABA_A_R and tonic inhibition, while blockade of GluN2B with ifenprodil led to an increase (Wu et al., 2021a). In contrast to moderate-to-high level NMDAR activation that destabilizes GABA_A_R, a low-level, brief NMDAR activation results in enhanced spontaneous IPSCs (Xue et al., 2011) and α5-GABA_A_R-mediated tonic current (Wyroślak et al., 2021) while simultaneously inducing eLTD (Rajgor et al., 2020). This NMDA-induced iLTP is further characterized by increased synaptic gephyrin accumulation and receptor insertion (Petrini and Barberis, 2014) and associated with the formation of subsynaptic nanodomains that stabilize IPSP amplitude (Pennacchietti et al., 2017). 90-min following NMDA stimulation, reduced microRNA-mediated gene silencing of α1- and γ2-GABA_A_Rs is responsible for increased surface receptor expression in iLTP, which occurs alongside enhanced silencing of the AMPAR GluA1 gene (Rajgor et al., 2020). NMDAR antagonism by APV blocks iLTP, confirming the impact of glutamatergic activity on inhibitory plasticity (Ouardouz and Sastry, 2000). In addition to ionotropic NMDARs, mGluR activation also regulates synaptic inhibition via inositol 1,4,5-trisphosphate receptor (IP3R)-dependent intracellular calcium store release and subsequent PKC-mediated stabilization of GABA_A_Rs at synapses (Bannai et al., 2015). Interestingly, eLTP is potentiated by pharmacological inhibition of mGluR or IP3R (Taufiq et al., 2005) or IP3R genetic deletion, likely due to the lower decay rate of calcium levels observed (Yoshioka et al., 2010).

### Effect of GABA Type A Receptor Signaling on Excitatory Synaptic Plasticity

Just as glutamatergic activity modulates inhibitory plasticity, GABA_A_R activity modulates excitatory plasticity. Pharmacological enhancement of GABA_A_Rs, as with the BZD flunitrazepam or 4,5,6,7-tetrahydroisoxazolo[5,4-c]pyridin-3-ol (THIP), a δ-GABA_A_R-preferring agonist, blocks eLTP (Seabrook et al., 1997; Whissell et al., 2013). Furthermore, potentiation of GABA_A_Rs by the anesthetics isoflurane, sevoflurane, and etomidate inhibits eLTP in a dose-dependent manner (Haseneder et al., 2009), where the effects of etomidate are mediated by β2-GABA_A_Rs (Figueroa et al., 2021). Several mechanisms for this dampening of excitation during potentiation of inhibition have been demonstrated. For example, isoflurane application for 6-h specifically enhances endocytosis of GluN2B-NMDARs (Dong et al., 2013), while co-application of muscimol with glutamate functionally reduces glutamate-induced calcium rise (Brady et al., 2018). Alcohol potentiates GABA_A_Rs and inhibits NMDARs (reviewed in Roberto and Varodayan, 2017). At physiologically relevant concentrations (Wallner et al., 2006; Olsen et al., 2007), a 30 min–1 h withdrawal from single-dose ethanol induces rapid subunit alterations in GABA_A_Rs. Most notably, extrasynaptic α4βδ-containing receptors are rapidly internalized, resulting in overall reduced tonic current and diminished ethanol-potentiation of tonic current (Liang et al., 2007; Shen et al., 2011; Suryanarayanan et al., 2011; Chen et al., 2018). Interestingly, α4 and γ2 subunit expression increase at later timepoints after withdrawal, beginning at 4-h and persisting for up to 48-h (Liang et al., 2007; Shen et al., 2011; Werner et al., 2011), potentially due to increased α4βγ2-GABA_A_Rs. A 15-min ethanol administration to hippocampal slices blocks tetanic-induced eLTP via a GABA_A_R-dependent mechanism (Ramachandran et al., 2015). Surprisingly, this increases expression of γ2-GABA_A_R along with AMPAR subunits GluA1 and GluA2, NMDAR subunit GluN2A, and PSD95. Ethanol treatment also increases production of allopregnanolone, a neurosteroid which potentiates GABA_A_Rs, contributing to the ethanol-dependent inhibition of eLTP (Ramachandran et al., 2015). While potentiating inhibition blocks eLTP, stifling inhibition with the GABA_A_R NAM DMCM (Seabrook et al., 1997) or the competitive antagonist bicuculline (Matsuyama et al., 2008) potentiates eLTP, which is associated with specific alterations in NMDAR expression. In immature DIV14 hippocampal neurons, 48-h inhibition of α5-GABA_A_Rs with the NAM L-655,708 decreased GluN2A, but increased synaptic GluN2B (Nuwer et al., 2021). Interestingly, as neurons mature to DIV21, L-655,708 had the opposite effect—synaptic GluN2A increased while synaptic GluN2B decreased. Additionally, increased neuronal activity uncouples α5-GABA_A_R from its extrasynaptic scaffold radixin (Hausrat et al., 2015), allowing α5-GABA_A_Rs to diffuse into inhibitory synapses, whereby increased synaptic α5 conductance prevents runaway LTP and freezes excitatory synaptic strength (Davenport et al., 2021).

### Interplay Between Synapses

Further supporting the interplay between glutamatergic and GABAergic plasticity, iLTP induced by low-frequency stimulation (LFS) causes eLTD, reducing EPSC amplitudes (Ravasenga et al., 2022). Using photo-stimulation to induce eLTP at a single spine, Ravasenga et al. (2022) further demonstrated differential plasticity of GABA_A_R synapses based on their relative spatial localization to the potentiating spine, such that inhibitory synapses <3 μm of the potentiating spine underwent iLTD and synapses >3 μm away underwent iLTP. These are examples of heterosynaptic plasticity, in which unstimulated synapses undergo plasticity in response to stimulation of a separate synapse. High-frequency glutamatergic stimulation at dendritic spines increases surface AMPARs at the potentiated synapse while nearby (<3.4 μm) unstimulated spines show a decrease in surface AMPARs and eLTD, with the degree of spine shrinkage inversely proportional to stimulated spine enlargement (Oh et al., 2015; Tong et al., 2021). Although these studies did not specifically examine inhibitory synapses, GABAergic inhibition can suppress bulk cytosolic Ca^2+^ increases and allow the preservation of NMDAR-generated Ca^2+^ nanodomains to induce spine shrinkage (Hayama et al., 2013) and control postsynaptic Ca^2+^ signals within an individual dendritic spine (Chiu et al., 2013). In support of this, heterosynaptic plasticity induced by spike time-dependent plasticity protocols is regulated by Ca^2+^-induced Ca^2+^ release to selectively adjust the synaptic strength from populations of inputs onto mouse auditory cortex (Field et al., 2020). Likewise, treatment with the group 1 mGluR agonist (S)-3,5-Dihydroxyphenylglycine (DHPG) triggers LTD at both excitatory and inhibitory synapses by distinct mechanisms in the lateral habenula (Valentinova and Mameli, 2016), and olfactory discrimination learning results in a CaMKII-dependent twofold balanced increase in GABA_A_R and AMPAR channel conductance in a subset of pyramidal cells in the piriform cortex (Reuveni et al., 2017). Simultaneous structural remodeling of inhibitory and excitatory synapses is also observed *in vivo* under different plasticity protocols. Monocular deprivation results in clustered remodeling of inhibitory synapses and dendritic spines within a restricted dendritic region of ∼10 μm (Chen et al., 2012). Interestingly, electron microscopy studies reveal that TBS-LTP spinogenesis in mature hippocampal CA1 dendrites consists of initial loss of small excitatory and inhibitory synapses with subsequent balanced enlargement of both synapses by 2-h (Bourne and Harris, 2011).

These studies collectively point to convergent glutamatergic and GABAergic signaling that allows activity-dependent receptor coordination for regulation and tuning of excitation/inhibition balance. Identifying crosstalking proteins and mechanisms that coordinate these signaling pathways are an important current and future area of investigation.

### Calcium Acts as a Master Regulator of Synaptic Crosstalk

**FIGURE 1:**
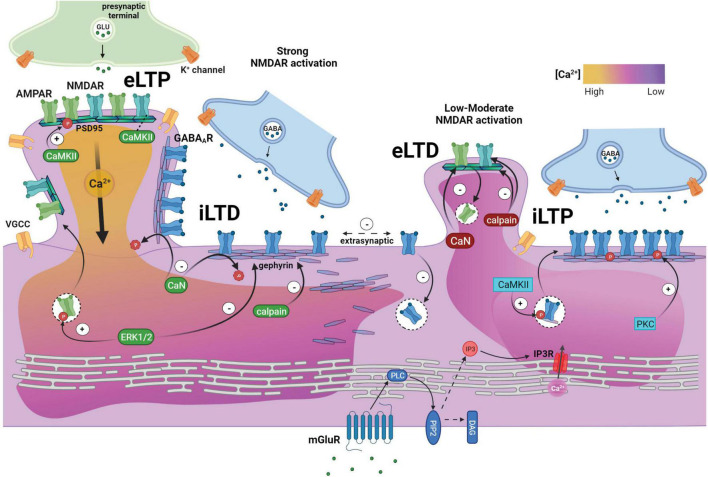
Relative local calcium levels facilitate glutamatergic and GABAergic synaptic crosstalk and plasticity responses through an intersection of downstream signaling pathways. **Left:** Strong glutamatergic activation and high calcium influx (yellow) triggers excitatory long-term potentiation (eLTP) associated with ERK1/2-mediated insertion of synaptic AMPARs, which is facilitated by translocation of CaMKIIα to excitatory synapses and interaction with NMDARs. CaMKIIα phosphorylation additionally stabilizes synaptic AMPARs. eLTP also induces heterosynaptic long-term depression of nearby inhibitory synapses (iLTD). During iLTD, calcineurin-mediated dephosphorylation of γ2-GABA_A_Rs increases receptor mobility and diffusion to extrasynaptic sites, while ERK1/2-mediated gephyrin phosphorylation and calpain protease activity disassembles the gephyrin scaffold. **Right:** During low-moderate NMDAR activation, CaMKIIα translocation to inhibitory synapses facilitates inhibitory long-term potentiation (iLTP) through synaptic gephyrin recruitment and enhanced β3-GABA_A_R forward trafficking and membrane insertion. Simultaneously, moderate NMDAR stimulation triggers a calcineurin-mediated reduction in surface AMPARs and calpain proteolytic degradation of NMDAR-GluN2B subunit and glutamatergic PSD95 scaffold (excitatory long-term depression, eLTD). eLTD and spine shrinkage is also observed in response to eLTP of proximal spines, while iLTP occurs at synapses distant from the potentiating spine. **Bottom:** Activation of group I metabotropic mGluR induces downstream IP3 receptor activation and release of calcium stores from the endoplasmic reticulum. The moderate increase in calcium concentration (pink) prompts PKC-mediated GABA_A_R phosphorylation and stabilization, contributing to the strengthening of inhibitory synapses during iLTP. Created with BioRender.com.

Considerable evidence supports divergent calcium signaling pathways as the primary mechanism for mediating crosstalk between excitatory and inhibitory synapses (Figure 1). Calcium entry via NMDARs plays a central role in excitatory synapse strengthening, offset by GABA_A_R-regulated dampening of Ca^2+^ signaling. Induction of iLTP showed dependence on glutamatergic NMDAR (Ouardouz and Sastry, 2000) or mGluR signaling (Morales-Weil et al., 2020), both of which trigger a rise in intracellular calcium, such that calcium chelation prevents iLTP (Ghafouri et al., 2019; Ravasenga et al., 2022). Bannai et al. (2015) demonstrate the presence of two distinct, non-overlapping mechanisms by which mGluRs and NMDARs trigger differential calcium-signaling pathways with opposing effects on hippocampal inhibitory synapses. In this model, group 1 mGluRs promote IP3R-dependent intracellular calcium store release from the endoplasmic reticulum, leading to PKC-mediated GABA_A_R stabilization. Conversely, NMDAR-induced calcium influx activates the phosphatase calcineurin, which dephosphorylates γ2-GABA_A_Rs at S327, resulting in enhanced GABA_A_R mobility required for the rapid induction of iLTD during elevated activity (Lu Y. M. et al., 2000; Wang et al., 2003; Muir et al., 2010; Bannai et al., 2015; Garcia et al., 2021). Pharmacological inhibition of NMDAR (APV), group 1 mGluR (MPEP and CPCCOEt), IP3R (Xestospongin C), or calcineurin (FK506) all prevent heterosynaptic eLTD, strongly supporting the role of these calcium signaling pathways in eLTD (Oh et al., 2015; Tong et al., 2021).

Voltage-gated calcium channels (VGCCs) permit calcium influx in response to membrane depolarization. This functional control over intracellular calcium makes VGCCs important contributors to multiple forms of plasticity at both synapse types (Nanou and Catterall, 2018; Gravielle, 2021). An overall dependence on NMDARs and L-VGCCs during homeostatic decreases in excitability is observed (Lee and Chung, 2014). Additionally, activation of L- and N-type VGCCs is necessary for LFS-induced iLTP. L-VGCCs also mediate heterosynaptic iLTD in response to single-spine eLTP (Ravasenga et al., 2022). Mechanistically, VGCCs can impact downstream receptor trafficking and receptor expression levels. L-VGCCs regulate GABA_A_R synaptic abundance by reducing proteosomal degradation and enhancing exocytosis of newly translated GABA_A_R (Saliba et al., 2009) via CaMKII phosphorylation of β3 at S383 (Saliba et al., 2012). On the other hand, L-VGCCs reduce nascent transcription of α1-GABA_A_Rs with 48-h DZP (Foitzick et al., 2020) and mediate enhanced GABA_A_R diffusion during chronic depolarization at the axon initial segment (Muir and Kittler, 2014).

Overall, these studies demonstrate the substantial impact that changes in local calcium concentrations can have on both glutamatergic and GABAergic synapses through the concerted action of key calcium-signaling proteins.

### Post-translational Modifications and Crosstalking Proteins in Synaptic Plasticity

PTM-dependent regulation of interactions between receptors, sub-membrane scaffolds, and other synaptic structural proteins is central to plasticity, with many synaptic-targeting kinases and phosphatases activated by rising intracellular calcium, including calcineurin, CaMKII, and PKC. The crosstalking protein discussion provided here focuses on gephyrin, CaMKII, KCC2, and calpain; see Table 1 for a more comprehensive list of proteins indicated in plasticity at excitatory and inhibitory synapses.

**TABLE 1:** Potential crosstalk proteins implicated in plasticity at both GABAergic and glutamatergic synapses.

Protein	General function	GABAergic synapses	Glutamatergic synapses	Both synapses
AKAP79^a^	AKAP79 in humans; AKAP150 in mice; scaffolding protein that anchors PKA, PKC, and CaN near the synaptic membrane (Klauck et al., 1996)	Brandon et al., 2003; Dacher et al., 2013; Carlson et al., 2014	Colledge et al., 2000; Tavalin et al., 2002; Lilly et al., 2005; Sanderson et al., 2016, 2018; Purkey et al., 2018; Woolfrey et al., 2018	Reviewed in Wild and Dell’Acqua, 2018
Calpain	Calcium-dependent cysteine protease; partially cleaves proteins to modulate protein function and/or localization	Tyagarajan et al., 2013; Costa et al., 2016	Andres et al., 2013; Baudry and Bi, 2016; reviewed in Wang et al., 2013	Reviewed in Wu and Lynch, 2006; Briz and Baudry, 2017
CaMKII	Serine/threonine protein kinase; Ca^2+^/calmodulin-dependent holoenzyme	Marsden et al., 2007, 2010; Saliba et al., 2012; Gao et al., 2014; Petrini et al., 2014; Ghosh et al., 2015; Morales-Weil et al., 2020; reviewed in Houston et al., 2009	Thalhammer et al., 2006; Patterson et al., 2010; El Gaamouch et al., 2012; Coultrap et al., 2014; Ghosh S. et al., 2016; Woolfrey et al., 2018; reviewed in Lisman et al., 2012; Khan et al., 2021	Oh et al., 2015; Reuveni et al., 2017; Cook et al., 2021; Tong et al., 2021
GRIP1^a^	PDZ domain-containing protein	Kittler et al., 2004; Li et al., 2005; Marsden et al., 2007	Dong et al., 1997; Kim et al., 2001; Takamiya et al., 2008; Mejias et al., 2011	Dong et al., 1999; Charych et al., 2004
KCC2	Regulates the neuronal Cl^–^ gradient by exporting Cl^–^ (Kaila et al., 2014)	Woodin et al., 2003; Huang et al., 2013; Heubl et al., 2017; Al Awabdh et al., 2022; reviewed in Cherubini et al., 2021	Wang et al., 2006; Gauvain et al., 2011; Chevy et al., 2015	Gulyás et al., 2001; Lee et al., 2011; Chamma et al., 2013; Sun et al., 2013; Garand et al., 2019; reviewed in Chamma et al., 2012
Np65^a^	Member of the immunoglobulin superfamily; brain- and neuron-specific cell adhesion molecule	Sarto-Jackson et al., 2012	Smalla et al., 2000; Empson et al., 2006	Herrera-Molina et al., 2014; reviewed in Beesley et al., 2014
NSF^a^	Member of the AAA + family of ATPases; involved in membrane trafficking and vesicle fusion (Furst et al., 2003)	Kittler et al., 2001; Goto et al., 2005; Marsden et al., 2007; Chou et al., 2010; reviewed in Luscher et al., 2011; Lorenz-Guertin and Jacob, 2018	Song et al., 1998; Beretta et al., 2005; Huang et al., 2005; Hanley, 2007; Araki et al., 2010; reviewed in Anggono and Huganir, 2012	
Pin1^a^	Catalyzes post-phosphorylation conformational modifications (Lu and Zhou, 2007)	Antonelli et al., 2014	Antonelli et al., 2016	
Shisa7^a^	Member of the CKAMP family; also called CKAMP59	Han et al., 2019; Wu et al., 2021b	Farrow et al., 2015; Schmitz et al., 2017	
SNX27^a^	Promotes recycling of PDZ-containing proteins to the plasma membrane (Lauffer et al., 2010)	Binda et al., 2019	Clairfeuille et al., 2016	

*^a^Proteins that are not discussed in the text.*

*AAA+, ATPases associated with diverse cellular activities; AKAP, A-kinase anchoring protein; CaMKII, Ca^2+^/calmodulin-dependent protein kinase II; CaN, Calcineurin; CKAMP, cystine-knot AMPA receptor–modulating protein; GRIP1, Glutamate Receptor Interacting Protein 1; KCC2, K + -Cl^–^ cotransporter 2; NL3, Neuroligin 3; Np65, Neuroplastin 65; NSF, N-ethylmaleimide-sensitive factor; PDZ, Post synaptic density protein, Drosophila disc large tumor suppressor, and Zonula occludens-1 protein; Pin1, Peptidyl-prolyl cis/trans Isomerase; SNX27, Sorting Nexin 27.*

GABA_A_R or NMDAR phosphorylation has distinct functional consequences depending on the subunit and site of phosphorylation (for review, see Chen and Roche, 2007; Nakamura et al., 2015). Gephyrin itself is also highly regulated by a multitude of PTMs (Zacchi et al., 2014; Ghosh H. et al., 2016; Battaglia et al., 2018). Chronic increases in activity (48-h 4AP treatment) result in gephyrin phosphorylation and synaptic accumulation, followed by a decrease in GABA_A_R diffusion (Battaglia et al., 2018). This contrasts with the effects of acutely increasing neuronal activity; here, gephyrin scaffold loss occurs subsequent to the increase in GABA_A_R diffusion triggered by receptor dephosphorylation (Bannai et al., 2009; Niwa et al., 2012). Using gephyrin phosphomutants, Battaglia et al. (2018) showed that chronic activity-induced phosphorylation of gephyrin by CaMKII, PKA, and GSK3β regulate synaptic GABA_A_R activity-dependent diffusion, while phosphorylation of gephyrin by GSK3β alone regulates extrasynaptic GABA_A_R activity-dependent diffusion. During acute periods of elevated activity, ERK1/2 phosphorylates gephyrin at S268 to activate calpain-mediated disassembly of the gephyrin scaffold and restrict inhibitory synaptic clustering (Tyagarajan et al., 2013). While ERK1/2 is not involved in activity-dependent regulation of gephyrin and GABA_A_R diffusion during chronic periods of elevated activity, it is involved in the activity-dependent regulation of extrasynaptic GABA_A_R mobility independent of gephyrin (Battaglia et al., 2018). Increased calpain-mediated gephyrin cleavage is also observed with 24-h DZP treatment (Lorenz-Guertin et al., 2019) and is necessary for iLTD of synapses close to spines undergoing eLTP (Ravasenga et al., 2022). Thus, gephyrin phosphorylation and calcium-activated calpain-mediated gephyrin cleavage are key regulators of inhibitory synaptic strength. Calpain also functions at excitatory synapses, where it similarly regulates PSD95 and GluN2B-NMDAR (Lu X. et al., 2000; Vinade et al., 2001; Hawasli et al., 2007; Doshi and Lynch, 2009).

CaMKII activation is required for both eLTP and eLTD (Coultrap et al., 2014; Cook et al., 2021), heterosynaptic plasticity (Oh et al., 2015; Tong et al., 2021), and iLTP (Morales-Weil et al., 2020). CaMKII also plays an important role in the long-lasting morphological changes in dendritic spines that coincide with the functional expression of plasticity (Matsuzaki et al., 2004; Lee et al., 2009). Both eLTP and eLTD/iLTP require CaMKII autophosphorylation at T286, while eLTD/iLTP requires subsequent CaMKII phosphorylation at T305/306 (Cook et al., 2021). Moderate NMDAR activation results in the translocation of CaMKIIα to inhibitory synapses (Marsden et al., 2010), which then recruits gephyrin to the synapse and increases GABA_A_R forward trafficking and synaptic stabilization through CaMKII-mediated phosphorylation of β3 at S383 (Petrini et al., 2014). Furthermore, earlier work by Marsden et al. (2007) revealed that moderate NMDAR activation can increase GABA_A_R membrane insertion via the activity of N-ethylmaleimide-sensitive factor (NSF), GABA_A_R-associated protein (GABARAP), and glutamate receptor interacting protein 1 (GRIP1). This same degree of activation also reduces surface AMPAR via activation of calcineurin and PP1 (Mulkey and Malenka, 1992; Beattie et al., 2000; Marsden et al., 2007). Conversely, strong NMDAR activation leads to CaMKIIα translocation to excitatory synapses (Strack et al., 1997; Shen and Meyer, 1999; Thalhammer et al., 2006) and increases AMPAR membrane insertion (Lemieux et al., 2012). Phosphorylation of AMPARs by ERK1/2, but not CaMKII, is required for activity-dependent AMPAR exocytosis (Patterson et al., 2010); interestingly, activation of GluN2B-containing, but not GluN2A-containing NMDARs results in ERK1/2 phosphorylation through a direct interaction between GluN2B and CaMKIIα (El Gaamouch et al., 2012).

Gephyrin scaffolding interactions with non-receptor proteins provides an additional mechanism of synaptic regulation. For example, gephyrin directly interacts with and regulates the surface expression of the neuronal chloride extruder K^+^-Cl^–^ cotransporter KCC2 (Al Awabdh et al., 2022). KCC2 is expressed near both inhibitory and excitatory synapses (Gulyás et al., 2001; Chamma et al., 2013) and is critical for generating the hyperpolarizing chloride gradient that allows for GABA_A_R inhibition in mature neurons. In addition to its chloride regulation function, KCC2 promotes dendritic spine development through a structural, ion-transport-independent manner via cytoskeletal interactions (reviewed in Virtanen et al., 2021). Increased activity reduces KCC2 clustering and promotes internalization via PP1-mediated dephosphorylation and calpain-dependent cleavage (Chamma et al., 2013), while enhanced GABA_A_R inhibition confines KCC2 to the plasma membrane via the Cl^–^ sensing kinase WNK1 (Heubl et al., 2017). Suppression of KCC2 precludes eLTP by preventing activity-dependent AMPAR membrane insertion in a KCC2 Cl^–^-transport-independent manner (Gauvain et al., 2011; Chevy et al., 2015). Interestingly, gephyrin may have further non-canonical function at excitatory synapses through interactions with neuroligins (NLGs), synaptogenic postsynaptic cell adhesion molecules which are implicated in cognition and contribute to autism (reviewed in Südhof, 2008). Co-immunoprecipitation experiments identified an association of gephyrin with both NLG2, exclusively expressed at inhibitory synapses, and NLG1, preferentially found at excitatory synapses (Varley et al., 2011). Further supporting evidence showed that hampering gephyrin function reduced VGLUT expression and mEPSC frequency.

In summary, subcellular localized domains for PTM-dependent regulation of receptors and other synaptic structural proteins allows for different forms of plasticity.

## Conclusion

Pharmacological manipulation of neuronal activity, largely targeted at GABA_A_R or NMDAR, has provided fundamental insights into mechanisms of glutamatergic and GABAergic synaptic plasticity. However, much remains to be understood on the interplay occurring between excitatory and inhibitory synapses. Outside of the postsynaptic molecular mechanisms described here, metabotropic GABA_B_Rs, glutamatergic kainate and AMPA receptors, presynaptic signaling, and local translation all contribute to the modulation of neuronal excitability and synaptic plasticity and are important areas of ongoing research for understanding synaptic crosstalk (see Castillo et al., 2011 for general iLTP/iLTD review). Further complexity is revealed upon investigating activity-induced changes in subpopulations of synapses that arise from different types of GABAergic interneurons (Yap et al., 2020; and reviewed in Chiu et al., 2019). Future investigations should strive to understand the concurrent impact of pharmacological manipulation and pathology on both excitatory and inhibitory transmission. In addition, more standardized treatment protocols for stimulation or inhibition of neuronal activity would facilitate cross-publication comparisons of results. Furthermore, few studies have investigated GABA_A_R plasticity with other GABA_A_R subtype-specific drugs, many of which are either current clinical therapeutics or are in development. Moving forward from examining receptor-focused plasticity events, identifying the localization and plasticity contributions of crosstalking proteins at each synapse type will be valuable. Although *in vivo* and slice studies are included throughout this mini-review, discussion focused on plasticity studied with primary rodent neuronal cultures. While this model is invaluable for elucidating molecular mechanisms at high resolution, neuronal cultures do not provide the same complex cellular, three-dimensional, and layer specific environment found with *ex vivo* or *in vivo* models. Thus, concerted efforts using cultured-neuron, slice, and *in vivo* studies will be needed to piece together a comprehensive understanding of the interplay between inhibitory and excitatory synapses under different states of neuronal activity or drug treatment. This knowledge will provide insights to target impaired synaptic plasticity in neurodevelopmental and neurological disorders.

## Author Contributions

CC, JN, and TJ wrote and edited the manuscript. CC prepared the figure. JN prepared the table with input from all authors.

## Conflict of Interest

The authors declare that the research was conducted in the absence of any commercial or financial relationships that could be construed as a potential conflict of interest.

## Publisher’s Note

All claims expressed in this article are solely those of the authors and do not necessarily represent those of their affiliated organizations, or those of the publisher, the editors and the reviewers. Any product that may be evaluated in this article, or claim that may be made by its manufacturer, is not guaranteed or endorsed by the publisher.
